# Editorial Notes

**Published:** 1933

**Authors:** 


### Editorial Notes

**Bristol Medical School.** THE centenary celebrations of the Bristol Medical School will take place on 30th June and 1st July. The programme of events is as follows:—

FRIDAY, JUNE 30TH.

| | |
|---|---|
| 3.30 p.m. | Receptions at the Bristol Royal Infirmary and the Bristol General Hospital by the Presidents. |
| 7 for 7.30 p.m. | Dinner at the University Union. |
| 9.30 p.m. | Reception at the University. |

SATURDAY, JULY 1ST.

| | |
|---|---|
| 12 noon. | Ceremony for Conferment of Ordinary and Honorary Degrees in the Great Hall. |
| 1.30 p.m. | Luncheon at the University Union. |
| 3.30 p.m. | Garden Party given by the Sheriff of Bristol, at the Royal Fort in the University. |

A history of the Medical School (which was founded in 1833, became the Medical Department of University College, Bristol, in 1879, and the School of Medicine of the University in 1909) has been prepared by Dr. George Parker. This history includes a complete list of alumni since 1833. It may be obtained from Messrs. John Wright & Sons, Bristol. Price 2s. 6d.

**Carey Coombs Memorial.** A COMMITTEE has been formed to promote a memorial to the late Dr. Carey Coombs, and an appeal for subscriptions is being circulated to his numerous friends. If any of these should fail to receive the appeal, subscriptions may be sent to Dr. H. Elwin Harris, jun., at 13 Lansdown Place, Clifton.

**Royal Society of Medicine.** ON Wednesday, 7th June, the Section of Surgery of the Royal Society of Medicine visited Bristol, and were entertained by the surgical staffs of the Royal Infirmary and of the General Hospital. A short account of the very full and varied programme appears on page 140.

